# Cubosomal functionalized block copolymer platform for dual delivery of linagliptin and empagliflozin: Recent advances in synergistic strategies for maximizing control of high-risk type II diabetes

**DOI:** 10.1007/s13346-023-01423-7

**Published:** 2023-10-08

**Authors:** Reham Waheed Hammad, Rania Abdel-Basset Sanad, Nevine Shawky Abdelmalak, Randa Latif

**Affiliations:** 1 Department of Pharmaceutics, Egyptian Drug Authority (formerly National Organization of Drug Control and Research (NODCAR)), Giza, Egypt; 2 Department of Pharmaceutics and Industrial Pharmacy, Faculty of Pharmacy, Cairo University, Kasr El Eini Street, Cairo, Egypt; 3 Department of Pharmaceutics and Industrial Pharmacy, School of Pharmacy, New Giza University, Giza, Egypt

**Keywords:** Linagliptin, Empagliflozin, Cubosomes, Diabetes mellitus, Non-linear pharmacokinetic

## Abstract

A well-made chitosan-PVP block copolymer platform was equipped with highly ordered and uniform nano-channels. This highly adhesive block copolymer platform was designed to ensure the efficient co-delivery of two synergistic-acting hypoglycemic drugs. Linagliptin oral bioavailability is 30% due to poor permeability and intestinal degradation. Its pharmacokinetics shows a non-linear profile. Empagliflozin exhibited decreased permeability and decreased solubility in aqueous media between pH 1 and 7.5. Cubosomes were functionalized as a good microdomain to guest and improve the physicochemical characteristics of drug molecules with decreased permeability and solubility. Cubosomes loaded with linagliptin (linagliptin cubosomes (LCs)) and empagliflozin (empagliflozin cubosomes ECs) were separately prepared using the top-down method and optimized by applying 2^3^ factorial design. Optimized cubosomal systems LCs (F3) and ECs (F4) were incorporated into a chitosan-PVP gel to obtain dual cubosome-loaded platforms (LECF) optimized through 2^2^ factorial design. The permeation study from the optimized LECF (C1) ensured enhanced empagliflozin permeation alongside continued efflux for linagliptin, resolving potential risks due to its non-linear plasma profile. The in-vivo study revealed that AUC_(0–∞)_ of linagliptin and empagliflozin was enhanced by 2- and threefold, respectively. Therefore, the chitosan-PVP block copolymer platform buccal application for the co-delivery of linagliptin and empagliflozin could contribute to enhanced clinical effectiveness in treating diabetes.

**Graphical Abstract:**

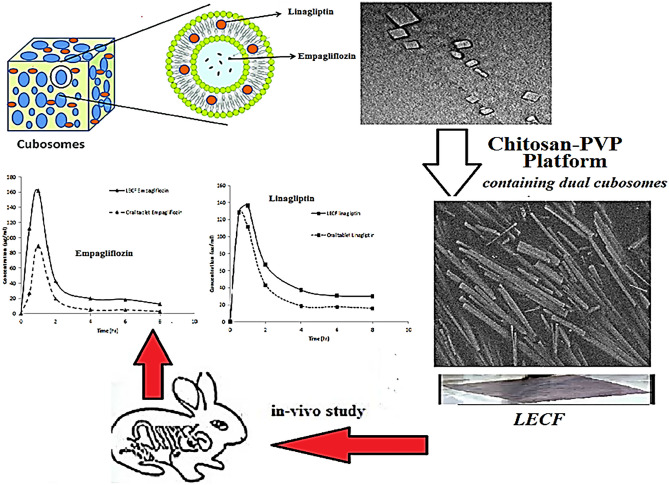

Graphical abstract showing dual cubosome-loaded platform tested in-vivo using a rabbit model

## Introduction

Type II diabetes mellitus (DM) is a metabolic disorder that leads to chronic impairment in the body’s glucose regulation and use. This results in excessive glucose circulating in the bloodstream (chronic hyperglycemia). Most patients diagnosed with diabetes (type II) for more than 5 years suffered from cardiovascular complications and showed signs of renal impairment. Anti-diabetic drugs like linagliptin (Lin) and empagliflozin (Emp) manage diabetes (type II) without hypoglycemia and cardiovascular risk factors [1, 2].

Lin competitively and reversibly inhibits the DPP-4 enzyme (enzyme causing inhibition of incretin hormones that stimulate insulin secretion from pancreatic beta cells in a glucose-dependent manner while inhibiting glucagon secretion from pancreatic beta cells) [3].

Emp selectively inhibits SGLT-2 (sodium-glucose co-transporter-2) that reabsorbs glucose from the glomerular filtrate in the kidney. This diminishes renal glucose reabsorption, encourages urinary glucose excretion, and diminishes plasma glucose concentrations [4].

Lin and Emp have synergistic mechanisms of action when co-administrated orally. They were registered to treat patients with type II diabetes [5]. Nevertheless, their co-delivery by oral route might not accomplish the desired effectiveness because of disadvantages in their pharmacokinetics. They show decreased permeability because they are class III in the BCS classification [6]. Emp is sparingly soluble in aqueous media between pH 1 and 7.5 [7]. Lin has a decreased oral bioavailability of 30% due to degradation by efflux-mediated P-glycoprotein existing in the intestine. [8]. The pharmacokinetics of Lin has non-linear profile outcomes from its slow dissociation from the enzyme DPP-4. [9]. Thus, the continuous efflux of Lin could nullify its non-linear pharmacokinetic profile. Lin molecules can be released in a continuous manner by formulating Lin cubosomes (LCs).

Cubosomes are self-assembled lipid bilayer systems that are twisted in an aqueous medium, forming three-dimensional structures with mixed hydrophilic and hydrophobic regions [10]. Their unique 3D nanostructure leads to a huge interfacial area which offers sustained diffusion paths and can provide increased encapsulation resulting from higher lipid concentration within the cubosomes [11]. This also permits the cubosomes to be loaded with poorly water-soluble drugs, such as Emp cubosomes (ECs) in their three-dimensional cubic phases that enhance their solubility, stability, and bioavailability [12]. Most investigated nanostructured cubosomal systems consist of monoolein (e.g., glyceryl monooleate) and water as binary systems. Pluronic F127 was the most pronounced hydrophilic surfactant used [10]. In this study isopropyl myristate (IPM) and phosphatidylcholine were utilized as novel candidates for preparing cubosomes that could incorporate lipophilic, hydrophilic, and amphiphilic drugs due to their viscous isotropic nature and large internal surface area.

The buccal mucosa is a useful route of trans-mucosal administration because it is easily accessible and contains an area of smooth muscle and relatively immobile mucosa. Therefore, it is suitable for administering controlled dosage forms. In addition, buccal drug delivery has a high degree of patient satisfaction compared to other trans-mucosal routes of drug administration, affords direct administration of drugs into the systemic circulation through the internal jugular vein, avoids acid hydrolysis in the gastrointestinal (GI) tract, and bypasses drugs from hepatic first-pass metabolism resulting in improved drug bioavailability [13].

Chitosan is a biodegradable, biocompatible polymer that possesses mucoadhesive properties, making it a potential candidate for buccal delivery. It could be useful in formulating cubosomal platforms to facilitate the buccal administration of cubosomes. Unfortunately, it significantly slows down the drug release [14], which is the most apparent inadequacy in formulating cubosomal platforms. This could be overcome by blending it with other polymers, such as PVP-K30 to improve drug release [15]. Chitosan-PVP platform enriched with Lin and Emp dual cubosomes could augment the bioavailability of the two drugs, enhance their clinical effectiveness in treating unresponsive hyperglycemic type II DM with associated cardiovascular disease side effects, and increase patient satisfaction.

## Materials and methods

### Materials

Lin and Emp were provided by ATCO Pharma and Apex Pharma, respectively. Other materials were baricitinib (IS) (BDR Life Science Private Limited), isopropyl myristate (IPM) (QUALIKEMS Fine Chemicals, Pvt, Ltd., India), glyceryl monooleate (GMO) and chitosan (MW 310000–375000 Da) (Sigma-Aldrich, U.S.A), L-alpha-lecithin (MW: 750.00 g/mol) (Acros Organics, USA), Kolliphor RH40 and Pluronic F127 (PF127) (Sigma-Aldrich, Germany), polyvinylpyrrolidone K30 (PVP K30) (Fluka Chem, Germany), glycerol (El-Nasr Pharmaceutical Chemicals, Egypt), and synthetic cellulose acetate membrane with a weight cut-off of 12,000–14,000 (SERVA Electrophoresis, Germany).

### Methods

#### Quantitative analysis of linagliptin and empagliflozin

The effective quantitative analysis of Emp and Lin concentrations was carried out according to the previously published method by Hammad et al. [16].

#### Experimental design

A full factorial design 2^3^ was used as shown in Tables 1 and 2, and experimental trials were performed at all sixteen formulae (eight formulae for Lin-loaded cubosomes (LCs) and eight formulae for Emp-loaded cubosomes (ECs)). The results obtained were analyzed using Design-Expert software (version 11; Minneapolis, USA) to find out the level of variables A, B, and C used in LC and EC preparation to get the desired criterion demonstrated in Table 1. ANOVA provision available in the software was utilized to validate the design of LCs and ECs and was adjusted at *p* < 0.05 [17].

**Table 1:** 2^3^ factorial design for linagliptin-empagliflozin cubosome (LC, EC) preparation (independent variables)

**Independent variables**	**Codes**	**Levels**	
Liquid lipid type	A	Isopropyl myristate	Glyceryl monooleate
Stabilizer type	B	Pluronic F127	Kolliphore
Lipid phase: stabilizer ratio (w/w)	C	9:1	7:3

**Table 2:** 2^3^ factorial design for linagliptin-empagliflozin cubosome (LC, EC) preparation (dependent variables)

**Dependent variables**	**Goal**
Particle size (nm)	Minimize
Percentage entrapment efficiency (%EE)	Maximize
Percentage release initially (%)	Maximize
Percentage release after 24 h (%)	Maximize

#### Preparation of linagliptin cubosomes (LCs) and empagliflozin cubosomes (ECs)

The top-down method was followed for the preparation of Lin-loaded cubosomes and Emp-loaded cubosomes as previously mentioned by Gaballa et al. [18] with modification. Top-down method is the most commonly used technique in the synthesis of cubosomes. This technique depends on the application of high levels of energy through high sheer homogenization in order to prepare the fine dispersion of cubosomes [19]. A specified amount of Lin (5 mg/mL) or Emp (10 mg/mL) was heated with the lipid mixture (GMO or isopropyl myristate: phosphatidylcholine (60:40) to 60 °C with magnetic stirring at 200 rpm (Jenway, LTD, UK) to make homogenous oil phase. The homogenous lipid phase was mixed with the aqueous phase containing PF127 or Kolliphor RH40 in different ratios, mentioned in Table 1, and then homogenized using a homogenizer (Ingenieurburo Zipperer GmbH, Germany) at 11.000 rpm for 10 min. The dispersion of cubosomes was kept in a glass container till further characterization.

Characterization of the prepared linagliptin cubosomes (LCs) and empagliflozin cubosomes (ECs) was performed as follows:

- Particle size (PS) and zeta potential (ZP) determination

The PS, polydispersity index (PDI), and ZP of the prepared LCs and ECs were measured by the dynamic light scattering method using Zetasizer 3000 (Malvern Instruments, UK).

- Entrapment efficiency (EE%) determination

Lin and Emp contents within cubosomes were separately quantified by the centrifugation method [20] for 2 h at 10,000 rpm at 0ºC. The quantity of unentrapped drugs in the supernatant was quantified by using HPLC. The entrapment percentage was calculated as follows [17]:

1 $$\mathrm{EE}\%=\frac{\mathrm{total\ drug}-\mathrm{free\ drug}}{\mathrm{total\ drug}}\times100$$

- In-vitro release study

The in-vitro release of Lin and Emp from LCs and ECs was carried out using the modified cylinder procedure [21]. Briefly, it was performed by placing 1 mL of LCs (containing 5 mg/mL Lin) and ECs (containing 10 mg/mL Emp) dispersions into a flat bottom cylinder with an internal diameter of 2 cm, their opening covered using a cellulose acetate, and fixing it onto a basket shaft of USP dissolution apparatus I. The modified cylinder was immersed into 300 mL phosphate-buffered saline pH 6.8 (according to the determined sink conditions) and rotated at 50 rpm at 37 ± 0.5 °C. Samples were substituted by an equal volume of fresh release medium and examined at predetermined time intervals (0.5, 1, 2, 3, 4, 6, 8, and 24 h). The statistical analysis was done for the average percent released of drugs from cubosome dispersions after 30 min (Q_30 min_) and after 24 h (Q_24 hr_). The data of drugs released from the prepared LCs and ECs were examined using different kinetic models to determine the release mechanism.

- Transmission electron microscopy and Fourier transform infrared spectroscopy (FTIR)

The optimized cubosomal systems (LCs and ECs) were studied by a transmission electron microscope (TEM) (JEM-100S, JEOL Ltd., Japan). FTIR (Agilent Cary 630, Germany) was utilized to obtain the Fourier transform infrared spectrum for linagliptin, empagliflozin, IPM, PC, and Kolliphor RH40 and for the optimized LCs and ECs [17].

#### Preparation of chitosan-PVP platform

The optimized LC- and EC-loaded chitosan-PVP platform (LECF) was prepared by adopting the solvent casting technique. The composition (as shown in Tables 3 and 4) and preparation of the prepared chitosan-PVP platform using the 2^2^ factorial design were presented in the previously published method by Hammad et al. [16]. The optimized LCs and ECs containing drugs were added to the polymer’s gel containing chitosan and PVP K-30 and stirred for 30 min. The prepared LC and EC chitosan-PVP platform was transferred into a glass mold of a diameter of 7 cm, placed into an oven (Memmert, Germany) at 45 °C, and cut into platforms of 4 cm^2^. A plain platform (PF) without drugs and a platform-containing powdered drugs (DF) were also prepared. The results obtained were analyzed using Design-Expert software to find out the level of independent variables used to obtain LECF with maximum drug content and maximum release initially and after 24 h for linagliptin and empagliflozin.

**Table 3:** Experimental plan of the factorial design 2^2^ for the preparation of chitosan-PVP platform

**Independent variables**	**Codes**	**Levels**	
Chitosan concentration	A	1%*	2%*
PVP-K30 concentration	B	70mg	150mg

^*^Percentage with respect to total formulation

**Table 4:** Experimental plan of the factorial design 2^2^ for the preparation of chitosan-PVP platform

**Dependent variables**	**Goal**
Drug content of linagliptin (%)	Maximize
Initial drug release of linagliptin (%)	Maximize
Release after 24 h of linagliptin (%)	Maximize
Drug content of empagliflozin (%)	Maximize
Initial drug release of empagliflozin (%)	Maximize
Release after 24 h of empagliflozin (%)	Maximize

Characterization and in-vitro/in-vivo studies of the chitosan-PVP platform were performed according the previously published method by Hammad et al. [16].

Characterization and evaluation of the buccal chitosan-PVP platform (LECF) was performed as follow**:**

- The thickness

Three randomly selected LECFs from each system were used for thickness determination using a micrometer (Hans Schmidt, GMBH, Germany).

- Weight uniformity of platforms

This was defined by measuring the weights of ten platforms of sizes 4 cm^2^ from each system and weighing them individually on an electronic balance.

- The surface pH

The buccal platform was permitted to swell by contact with distilled water (1 mL) for 1 h at room temperature. The pH was measured by bringing the electrode of the pH meter (Jenway Ltd., UK) in contact with the surface of the platform and permitting it to equilibrate for 1 min.

- Folding endurance value

The value was obtained from the number of times the platform could be folded mechanically at the same place without breaking or at least up to 300 times manually. Its value indicates the extent of flexibility of the platforms that are necessary for handling.

- Drug content

Drug content was determined by dissolving the platform (4 cm^2^), by sonication, in 100 mL of distilled water containing 1.5% v/v acetic acid for 5 h. After filtration through a filter (0.45 μm) to eliminate the insoluble residue, 5 mL of the filtrate was diluted to 10 mL with diluent. The drug content was then assessed using HPLC.

- Swelling index (SI)

The measurement was done to determine the extent of hydration of the hydrophilic polymers used in the platform’s preparation, which is necessary to initiate intimate mucosal surface contact. The SI measurement of the platforms was conducted according to the method previously published by Deshmane et al. (2009).

- Tensile strength (TS) study

It is defined as the total weight, which is necessary to break or rupture the platform. The 4 cm^2^ buccal platform from each formulation was fixed between the stationary and movable plate of the tensile strength tester (Tinius Olsen, USA). The force needed to break the platform was measured.

- Mucoadhesive strength measurement

The mucoadhesion strength between the platforms and mucosa membrane excised from sheep buccal mucosa was determined using a modified physical balance identical to that previously published by Pendekal and Tegginamat [22].

- In-vitro release study of linagliptin and empagliflozin from different LECF systems

The release was performed according to Deshmane et al. [15] using the same condition prescribed in the in-vitro release study of LCs and ECs. The platform of 4 cm^2^ (containing 5 mg linagliptin and 10 mg empagliflozin) was immersed (using supported weights) in the vessel of USP dissolution apparatus type II (Agilent, USA). Samples were assessed by HPLC. The statistical analysis was conducted for the percentage drug release of linagliptin and empagliflozin from the prepared LECF.

#### Optimization and morphology of the optimized cubosomal platform (LECF)

The results of the experimental design were analyzed using Design-Expert software [17] to find out the level of independent variables that would produce the desired criterion illustrated in Table 3. The morphology of the optimized LECF was examined utilizing scanning electron microscopy (Quanta FEG 250, Japan).

#### Ex vivo permeation study

Linagliptin and empagliflozin permeation across sheep buccal mucosa from the optimized LECF and the platform-containing powdered drugs (DF) were studied. Buccal sheep mucosa was obtained from the local slaughterhouse and used within 2 h. The test was carried out using a modified dissolution apparatus similar to that described by Hammad et al. [17]. The platform-containing powdered drugs (DF) and optimized LECF (each containing 5 mg of linagliptin and 10 mg of empagliflozin) were added to the surface of the mucosa in the donor compartment. The receptor compartment (containing 100 mL of PBS pH 6.8) was maintained at 37 ± 0.5 °C and stirred at 50 rpm. Samples from the receptor compartment were evaluated by HPLC at different time intervals. The averages of linagliptin and empagliflozin cumulative amounts permeated through sheep buccal mucosa per unit surface area were calculated and plotted as a function of time.

#### Pharmacokinetic study

The Institutional Ethical Committee (Faculty of Pharmacy, Cairo University) reviewed and approved the animal study protocol (PI2690). Six white male New Zealand rabbits (2.25–2.75 kg) were housed on a 12-h light/12-h dark cycle in an animal housing facility and provided access to food and water. The rabbits were randomly scattered into two groups. The first one received the optimized LECF; the second received the commercial oral tablet. A cross-over design was followed after the 7-day washing-out period. Rabbits were fasted overnight with free access to water and then anesthetized for 4–5 h using an intramuscular injection of ketamine [23]. LECF (1 cm^2^ according to animal dose adjustment calculation) was slightly wetted using 30 µL of water and applied to the buccal mucosa of the rabbits.

Following the buccal application of the optimized LECF and orally administrated tablet, blood samples were withdrawn from a cannula introduced into the marginal ear vein and evacuated into heparinized tubes immediately at (0.5, 1.0, 2.0, 4.0, 6.0, 8.0, and 24.0 h) following the buccal application of the optimized LECF and orally administrated tablet. Plasma was separated once blood samples were centrifuged at 5000 rpm for 10 min and kept frozen at – 70 °C until drug analysis.

The assay of linagliptin and empagliflozin in rabbit plasma samples was done using a validated, sensitive, and reliable HPLC method [24]. Thermo Scientific HPLC system comprised different flow rates (model Dionex 3000 series, USA), equipped with a variable wavelength detector set at 218 nm. The stationary phase was the Promosil C18 (250 × 4 mm) column. The mobile phase (acetonitrile: 0.02 M potassium dihydrogen phosphate buffer pH 3.5 adjusted with O-phosphoric acid) (35:65) was pumped at 1 mL/min at 25 °C. Baricitinib was used as an internal standard (ISD); the linagliptin, ISD, and empagliflozin were eluted at about 3.6, 4.5, and 6 min, respectively. Plasma (250 μL) containing 10 μL baricitinib solution (0.5 mg/mL) as IS was mixed with 2 mL acetonitrile and centrifuged at 10,000 rpm for 15 min. One milliliter of the supernatant was diluted with 1 mL diluent (water: acetonitrile 1:1) and assessed by HPLC. The linearity, precision, selectivity, and accuracy of the method were established before the assay.

Statistical analysis was executed using a statistical software program (SPSS Inc., Chicago, USA). The differences in the average data were compared by a simple analysis of variance (one-way ANOVA) in the software. The significance of the difference was determined at a 95% confidence limit (α = 0.05).

#### Development of in-vitro/in-vivo correlation

The relationship between in-vitro release data and in-vivo drug absorbed from the optimized LECF was proven by investigating plasma concentration data from pharmacokinetic studies [17], using a level A correlation which signifies a point-to-point correlation [17] between in-vitro release data and in-vivo absorption. The percentage of linagliptin and empagliflozin absorbed as a function of time was calculated using the Wagner–Nelson method similar to that described by Hammad et al. (2018).

#### Buccal histopathology

Histopathological evaluation was carried out on the sheep buccal mucosa to ensure the safety of LECF formulation [25]. Three pieces of sheep buccal mucosa with even thickness were selected and treated with phosphate-buffered saline pH 6.8 (negative control), optimized LECF, and isopropyl alcohol (positive control) respectively for 2 h. Subsequently, the mucosa was preserved in 10% v/v formalin solution overnight. The pieces of sheep buccal mucosa were introduced into paraffin blocks and cut by a microtome into sections. Sections were stained with hematoxylin–eosin and examined under the optical microscope (Olympus Tokyo, Japan). Photomicrographs were taken using a digital camera (Olympus, DXC-1850P, Tokyo, Japan) to estimate any changes that occurred to the sheep buccal mucosa.

## Results and discussion

### Quantitative analysis of linagliptin and empagliflozin

**Fig. 1:**
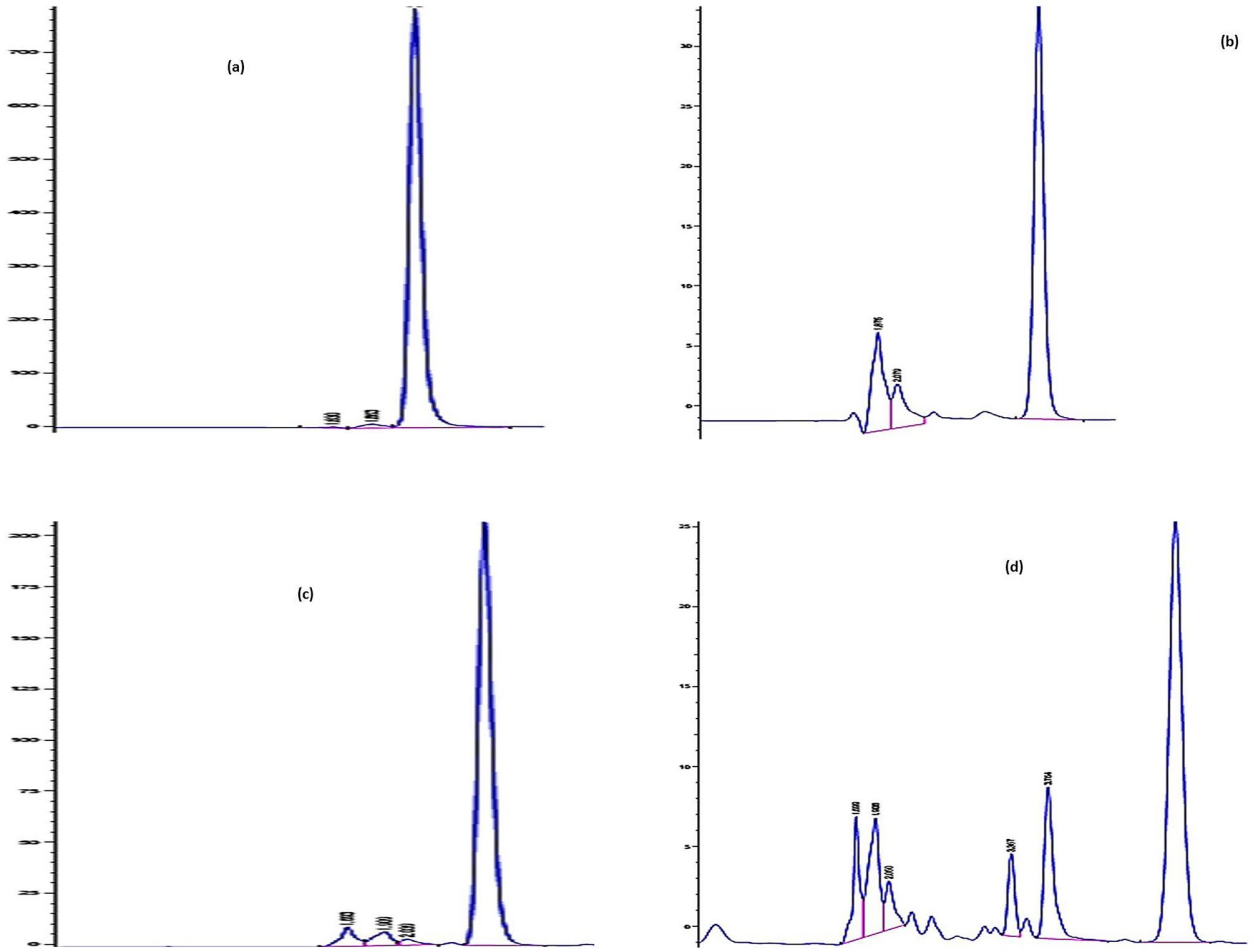
**a** HPLC chromatogram of linagliptin standard solution in diluent (methanol: water (1:1)). **b** HPLC chromatogram of empagliflozin standard solution in diluent (methanol: water (1:1)). **c** HPLC chromatogram of linagliptin standard solution in PBS at pH 6.8 measured at 254 nm. **d** HPLC chromatogram of empagliflozin standard solution in PBS at pH 6.8 measured at 254 nm

The quantitative analysis of Emp and Lin was done according to Hammad et al.’s. method [16] (as shown in Fig. 1). The verification was conducted as mentioned in USP general chapter verification requirements (1226).

### Preparation of linagliptin-loaded cubosomes (LCs) and empagliflozin-loaded cubosomes (ECs)

The top-down method was used to formulate LCs and ECs according to the composition mentioned in Table 1 with specific ratios of GMO or IPM: phosphatidylcholine (60:40) (the main forming lipid). IPM (C_17_H_34_O_2_) consists of esters of propan-2-ol and saturated high molecular weight fatty acids, principally myristic acid. GMO is a mixture of glycerides of oleic acid and other fatty acids, consisting mainly of the monooleate (C_21_H_40_O_4_). Here, we used phosphatidylcholine (with GMO and IPM) which is more amphipathic (both hydrophobic and hydrophilic) in aqueous media. This fact might increase the critical entrapment parameter obtained upon solubilization of phosphatidylcholine and lead to the formation of cubosomes [26, 27]. PF127 and Kolliphor RH40 were the stabilizers used in cubosome preparation. Regarding the PF127, it is a triblock copolymer composed of the hydrophobic domain (PPO) which is aligned parallel to the lipid bilayer of cubosomes, and the hydrophilic (POE) domains outspread into the aqueous milieu providing steric shielding, thereby preventing aggregation of the formed cubosomes [18]. Similarly, it was suggested that Kolliphor RH40 could be considered as an alternative stabilizing agent as it possessed structural similarity with it [28]. Kolliphor RH40 is composed of glycerol polyethylene glycol oxystearate, which, together with fatty acid glycerol polyglycol esters, formed the hydrophobic component of the stabilizer. The hydrophilic component is composed of polyethylene glycols and glycerol ethoxylate. The hydrophobic domain could be accommodated by the lipid bilayer leaving the long hydrophilic chain facing the aqueous milieu. Additionally, Kolliphor RH40 could afford a steric stabilizing effect preventing vesicle aggregation [18]. The following polynomial equations were generated using the Design-Expert software for analysis of the results:

**Table:** 

**EE%(LCs)**	= + 54.97 + 2.50A − 4.06B − 4.40C − 0.65AB + 0.53AC − 5.09BC + 1.08ABC	**(3)**
Q_30 min_ (LCs)	= + 34.97 − 5.44A − 4.90B − 16.73C + 8.83AB − 2.17AC − 4.71BC + 6.39ABC	**(4)**
Q_24 h_ (LCs)	** = + **69.07 + 0.36A − 5.40B − 4.94C + 4.63AB + 5.06AC − 4.32BC + 5.39ABC	**(5)**
EE%(ECs)	** = + **83.52 + 0.13A − 1.89B + 2.43C − 1.31AB − 1.52AC + 0.36BC − 0.74ABC	**(6)**
Q_30 min_ (ECs)	** = + **21.94 − 1.92A + 11.96B − 4.76C + 3.09AB − 1.27AC − 6.02BC − 3.24ABC	**(7)**
Q_24 h_ (ECs)	** = + **71.22 + 9.38A + 8.34B + 2.72C − 4.72AB − 1.75AC − 0.15BC − 2.87ABC	**(8)**

### Characterization of prepared LCs and ECs

Measuring the size of cubosomes is very essential due to their effects on the physical stability of the prepared formulations. PS also has a critical importance in controlling the cubosome penetration through the mucosal membrane, thus affecting the bioavailability of the loaded drug. All the prepared systems were in nano-size (Table 5). The PDI results were < 1 for all cubosomes, indicating dispersion homogeneity. The high magnitude of ZP of the prepared cubosomes indicated their stability. The ZP obtained for all formulations was negative values (Table 5). This could be due to the existence of ionized lipid moieties that have a major contribution to their electrostatic stabilization [29].

**Table 5:** Particle size, PDI, zeta potential, and entrapment efficiency of linagliptin-loaded cubosomes (LCs) and empagliflozin-loaded cubosomes (ECs)

**Formula**	**Particle size (nm)**	**PDI**	**Zeta potential (mV)**	**EE%**
F1 (LCs)	231 ± 27.6	0.3835 ± 0.09	−38.3 ± 0.07	54.21 ± 0.78
F2 (LCs)	236.6 ± 29.1	0.428 ± 0.09	−38.2 ± 0.64	57.40 ± 0.67
F3 (LCs)	151.9 ± 18.2	0.423 ± 0.07	−49.1 ± 0.53	60.34 ± 0.92
F4 (LCs)	119 ± 0	0.3555 ± 0.02	−41.4 ± 0.11	37.06 ± 1.55
F5 (LCs)	115.2 ± 0.7	0.199 ± 0.02	−35.8 ± 0.78	62.02 ± 0.03
F6 (LCs)	120.4 ± 0.4	0.187 ± 0.01	−26.7 ± 0.28	62.18 ± 0.66
F7 (LCs)	108.6 ± 0.8	0.3185 ± 0.03	−27.6 ± 0.3	60.62 ± 0.39
F8 (LCs)	127 ± 1.2	0.377 ± 0.01	−38.5 ± 0.26	44.86 ± 0.24
F1 (ECs)	348.5 ± 25.4	0.495 ± 0.03	−34.5 ± 0.42	81.09 ± 0.08
F2 (ECs)	233.5 ± 25.2	0.3645 ± 0.03	−31.5 ± 0.4	86.82 ± 0.04
F3 (ECs)	153.4 ± 20.2	0.2865 ± 0.001	−27.3 ± 0.98	77.84 ± 0.18
F4 (ECs)	128.6 ± 2.1	0.276 ± 0.001	−26.7 ± 0.42	87.85 ± 0.14
F5 (ECs)	164.9 ± 1.3	0.269 ± 0.004	−34 ± 0.28	85.53 ± 0.13
F6 (ECs)	158.1 ± 0.6	0.254 ± 0.004	−31.5 ± 0.68	88.14 ± 0.12
F7 (ECs)	128.2 ± 1.8	0.259 ± 0.01	−32.7 ± 0.42	89.16 ± 0.06
F8 (ECs)	128 ± 0.3	0.2855 ± 0.002	−36.5 ± 0.83	80.99 ± 0.21

## Entrapment efficiency determination

**Fig. 2:**
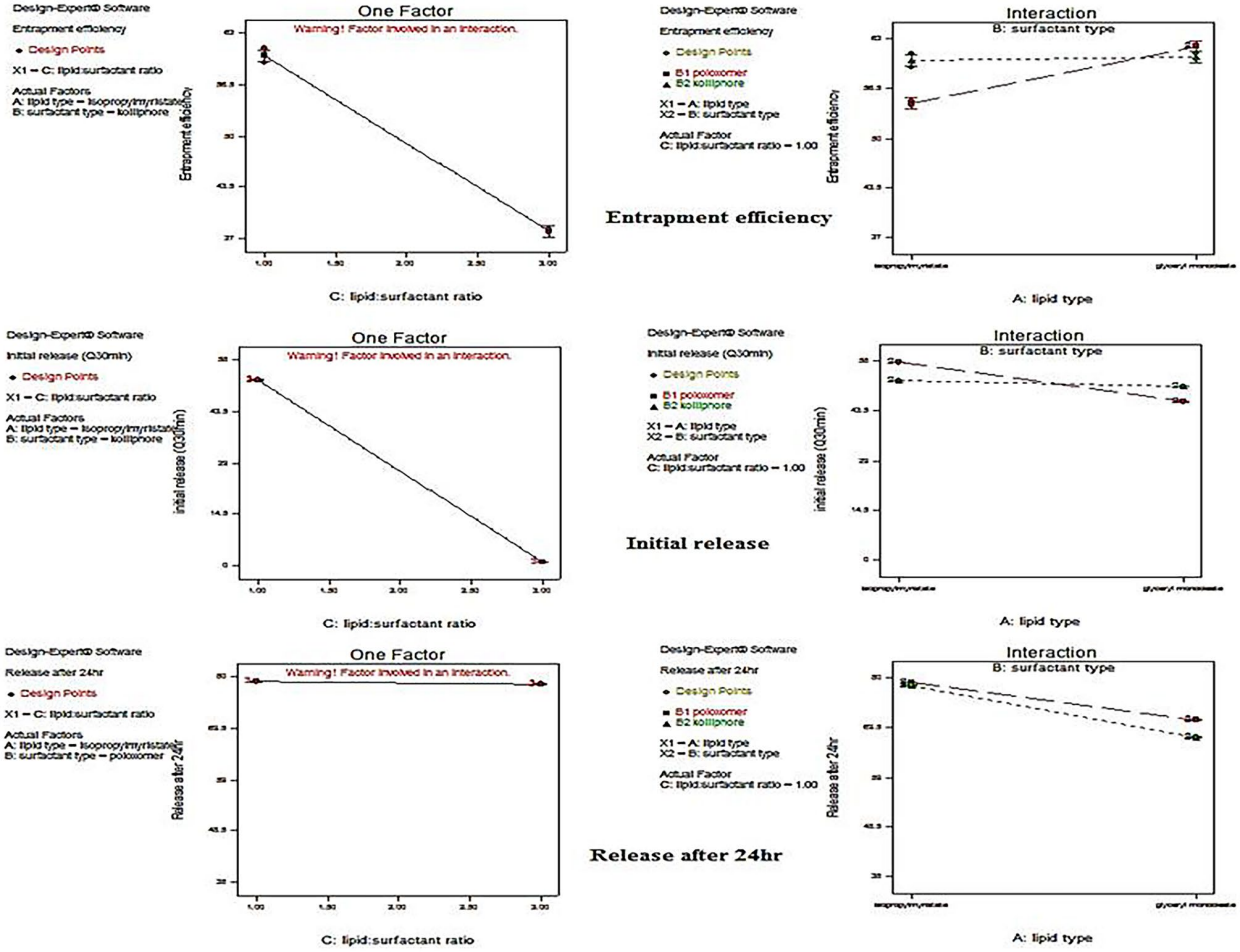
One-factor plot showing the effect of lipid-to-surfactant ratio (C) and interaction plot for the effect of liquid lipid type A versus surfactant type B on the mean dependent variable (entrapment efficiency, initial release, and release after 24 h) of linagliptin for the prepared linagliptin cubosomes (LCs)

**Fig. 3:**
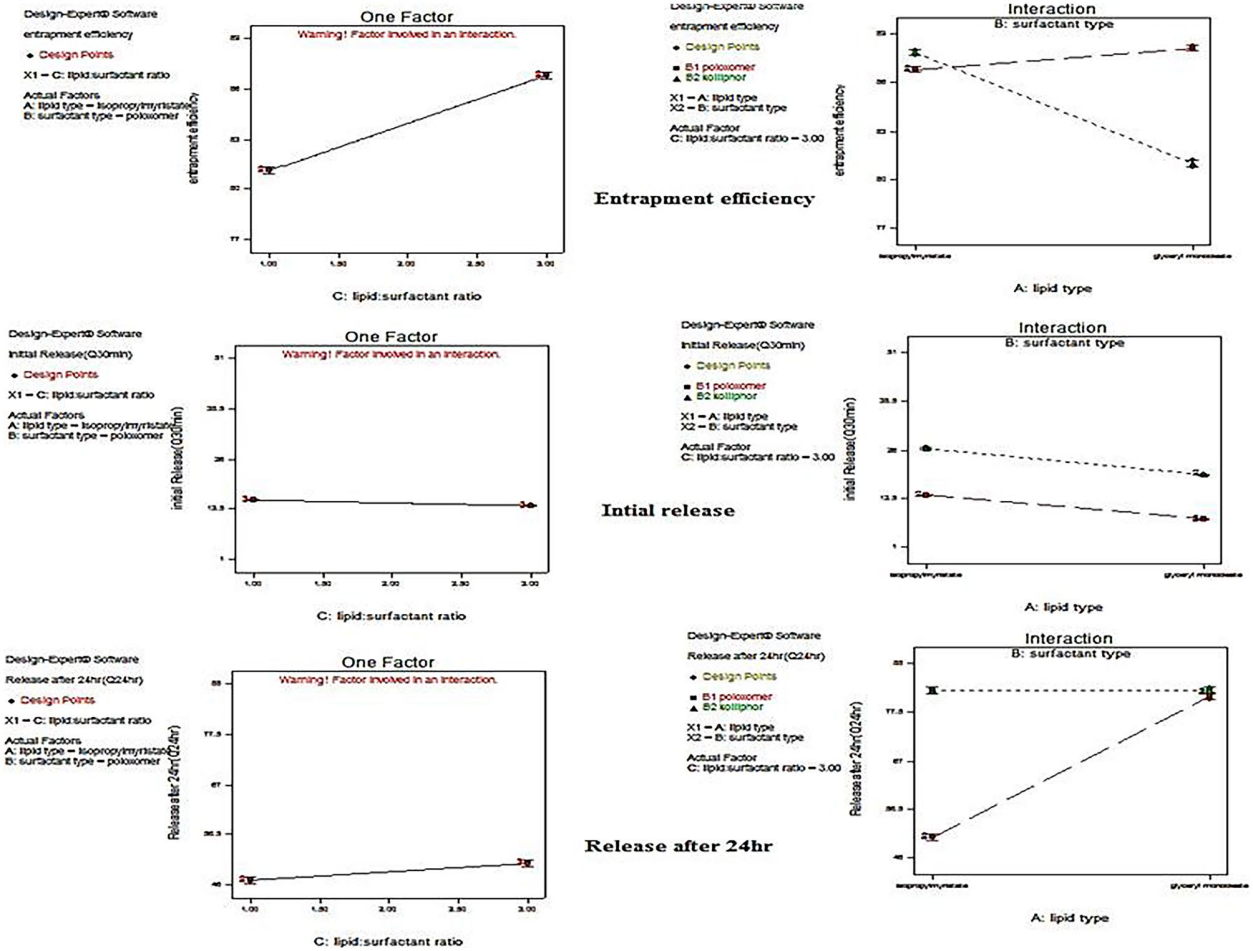
One-factor plot showing the effect of lipid-to-surfactant ratio (C) and interaction plot for the effect of liquid lipid type A versus surfactant type B on the mean dependent variable (entrapment efficiency, initial release, and release after 24 h) of empagliflozin for the prepared empagliflozin cubosomes (ECs)

The highest coefficient value of the factor lipid phase: stabilizer ratio (C) confirmed that it was the main factor which significantly affects the EE% of Lin and Emp within the prepared cubosomes (as shown in Figs. 2 and 3 and in Eqs. (3) and (6)).

### EE% of linagliptin in the prepared linagliptin cubosomes (LCs)

The one-factor plot emerged during the statistical analysis for the effect of lipid phase-to-stabilizer ratio (C), and the negative value of C in Eq. (3) indicated that increasing the concentration of surfactant resulted in decreasing the EE% of Lin. The best-used lipid phase-to-stabilizer ratio (C) was 9:1. This could be attributed to the fact that it belongs to class III drugs and is soluble in aqueous media. Analyzing the other factors (liquid lipid type A and surfactant type B) using the interaction plot that emerged during the statistical analysis indicated that IPM and Kolliphor RH40 improved the EE% of linagliptin compared to PF127, and GMO at lipid phase-to-stabilizer ratio (C) was 9:1. This was accounted to the lower HLB value of Kolliphor® RH40 if compared to that of PF127. The HLB value of Kolliphor RH40 lies between 14 and 16, while the HLB value of PF127 is 22. It is noteworthy that the HLB value of IPM was 11.5, higher than the GMO HLB value of 4.2 [30]. The interaction plot showed that both IPM and GMO did not have an effect on the EE% of Lin when formulating LCs using Kolliphor RH40 as a surfactant and using a lipid phase-to-stabilizer ratio (C) (9:1).

### EE% of empagliflozin in the prepared empagliflozin cubosomes (ECs)

The one-factor plot emerged during the statistical analysis for the effect of the lipid phase-to-stabilizer ratio (C), and the positive value of C in Eq. 6 indicated that increasing the concentration of surfactant resulted in increasing the EE% of Emp. The best-used lipid phase-to-stabilizer ratio (C) was 7:3. This could be attributed to its sparingly soluble nature in aqueous media between pH 1 and 7.5 [7]. Analyzing the other factors, liquid lipid type A and surfactant type B using the interaction plot that emerged during the statistical analysis indicated that the lipid phase-to-stabilizer ratio (C) was 7:3, using IPM with Kolliphor RH40 had similar EE% to that obtained when using GMO with PF127.

## In-vitro drug release

**Fig. 4:**
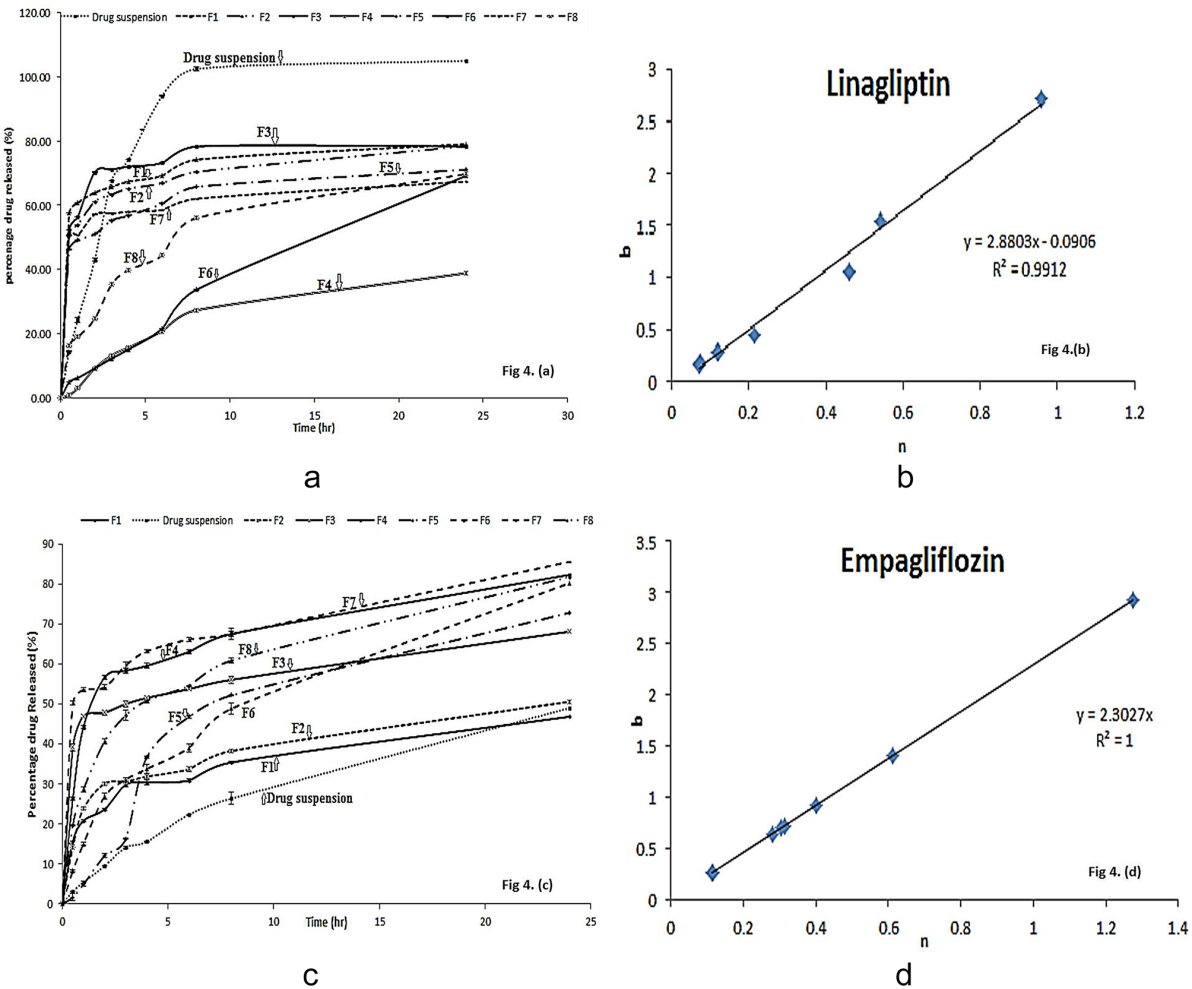
**a** In-vitro release percentage of linagliptin from LCs. **b** Linear regression of the estimates for “*b*” of the Weibull model versus the estimates for “*n*” of the Korsmeyer-Peppas model for linagliptin release from LCs. **c** In-vitro release percentage of empagliflozin from ECs. **d** Linear regression of the estimates for “*b*” of the Weibull model versus the estimates for “*n*” of the Korsmeyer-Peppas model for empagliflozin release from ECs

The in-vitro drug release was done separately for LCs and ECs cubosomal formulations. The results are demonstrated in Fig. 4a, c.

### Release of linagliptin in the prepared linagliptin cubosomes (LCs)

The one-factor plot emerged during the statistical analysis for the effect of lipid phase-to-stabilizer ratio (C), and the highest negative value of C as shown in Figs. 2 and 3 and in Eq. (4) demonstrated that the lipid phase-to-stabilizer ratio (C) was significantly affecting the release after 30 min (Q_30 min_) of Lin. The best-used lipid phase-to-stabilizer ratio (C) was 9:1. Analyzing the other factors (liquid lipid type A and surfactant type B) using the interaction plot that emerged during the statistical analysis indicated that IPM improved the initial release (especially when using PF127 as a surfactant) and the release after 24 h (Q_24 h_) of linagliptin at lipid phase-to-stabilizer ratio (C) (9:1). This was mainly due to its higher HLB value than GMO.

### Release of empagliflozin in the prepared empagliflozin cubosomes (ECs)

The one-factor plot emerged during the statistical analysis for the effect of lipid phase-to-stabilizer ratio (C) demonstrated that the lipid phase-to-stabilizer ratio (C) was significantly affecting the release after 30 min (Q_30 min_) of Emp. The best-used lipid phase-to-stabilizer ratio (C) was 7:3. Analyzing the other factors (liquid lipid type A and surfactant type B) using the interaction plot that emerged during the statistical analysis indicated that IPM improved the initial release and the release after 24 h (Q_24 h_) of empagliflozin at lipid phase-to-stabilizer ratio (C) 7:3. This was mainly due to its higher HLB value than GMO. Kolliphor RH40 resulted in higher initial release and release after 24 h (Q_24 h_) of empagliflozin. This accounted for the tendency of micelle formation upon the addition of PF127 (triblock copolymer containing both hydrophilic (PEO) chains and hydrophobic (PPO) chains) with lipid mixture including phosphatidylcholine. These micelles could entrap the hydrophobic drug (empagliflozin) within the micellar core retarding its release, while Kolliphor RH40 exerted a lower tendency of micelle formation upon its addition to lipid mixture including PC, leading to higher solubilization effects and higher empagliflozin release [31].

### Release kinetic of linagliptin and empagliflozin from the prepared cubosomes

It was previously published that the modeling of drug release from different delivery systems helps with the prediction of drug transport mechanisms [32]. The release profiles from LC and EC formulations were fitted to zero-order, first-order, Higuchi [33], Korsmeyer-Peppas [34], and Weibull models. The most appropriate model was selected depending on the best goodness-of-fit *R*^2^ values. The release from LC and EC formulations followed the Weibull model with coefficients *R*^2^ ranging from 0.903 to 0.99 for LCs and from 0.857 to 0.98 for ECs. Papadopoulou et al. [32] offered that the exponent of time *b* of the Weibull function is linearly interrelated to the exponent *n* of the power-law resulting from the Korsmeyer-Peppas equation [32]. The estimates of *b* are plotted versus the estimates of *n* for all data analyzed (as shown in Fig. 4b, d). The linear relationship established indicates the mathematical relevance of the exponents “*b*” of the Weibull model and exponents “*n*” of the Korsmeyer-Peppas equation, but the Weibull model is a valuable detective tool for a controlled release mechanism [35, 36]. The values of exponent *b* indicated a complex release mechanism [32] from different LCs and ECs due to 3D cubosome matrix system geometry.

### Optimization of the prepared LCs and ECs

The Design-Expert software selected the optimized LC (F3) system (prepared by IPM as liquid lipid (A), Kolliphor RH40 as a stabilizer (B), with lipid phase-to-stabilizer ratio (C) 9:1) and EC (F4) system (prepared by IPM as liquid lipid (A), Kolliphor RH40 as a stabilizer (B), with lipid phase-to-stabilizer ratio (C) 7:3) according to the desired criterion in Table 1.

### Morphology of the prepared LCs and ECs

**Fig. 5:**
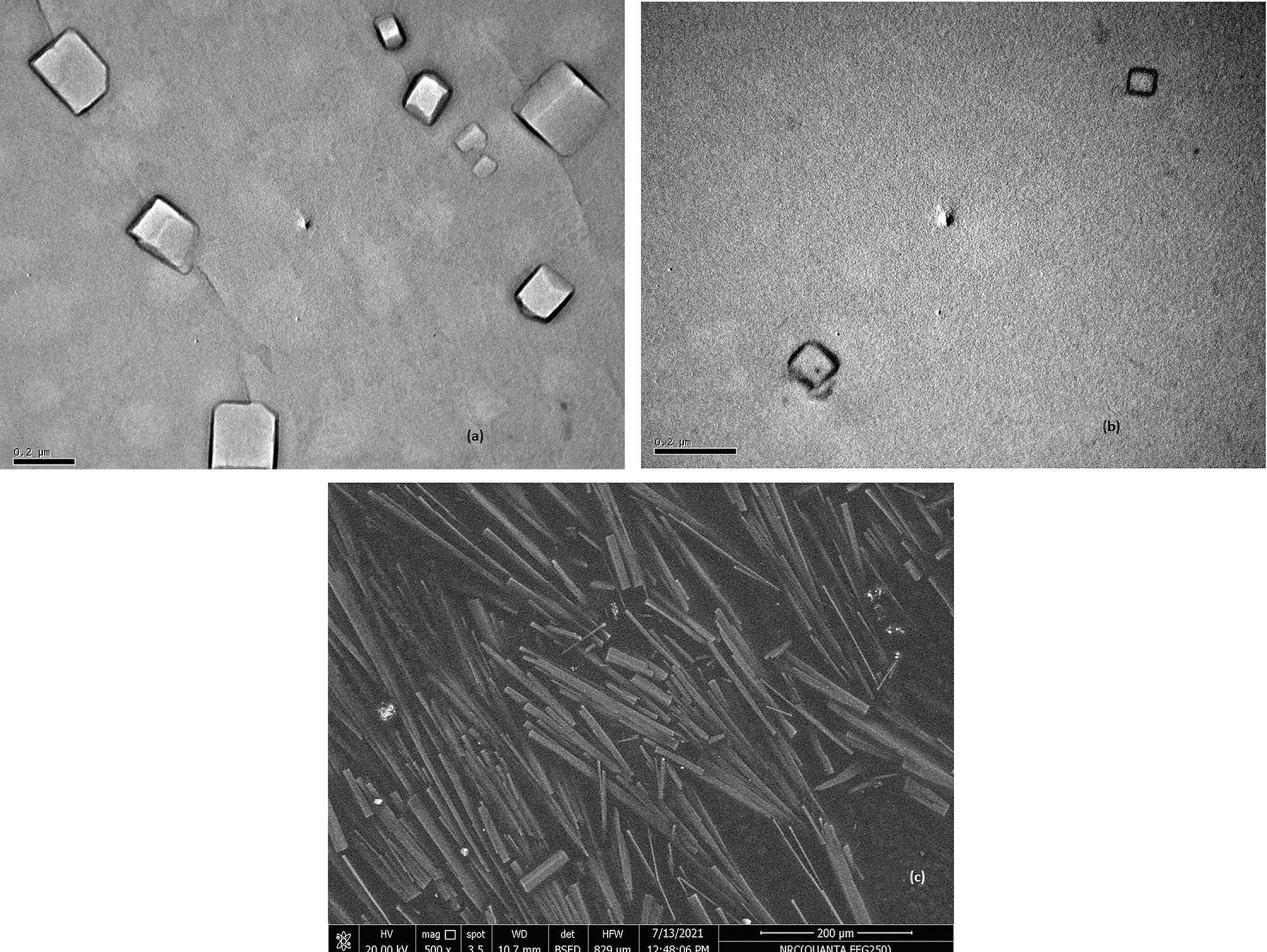
**a** TEM image of the optimized linagliptin cubosomes (LCs) (F3). **b** TEM image of the optimized empagliflozin cubosomes (ECs) (F4). **c** SEM image of the optimized cubosomal film (LECF) (C1)

TEM images (Fig. 5a, b) ensured the formation of cubosomes with their cubic configuration and 3D structure.

### Fourier transform infrared spectroscopy (FTIR)

**Fig. 6:**
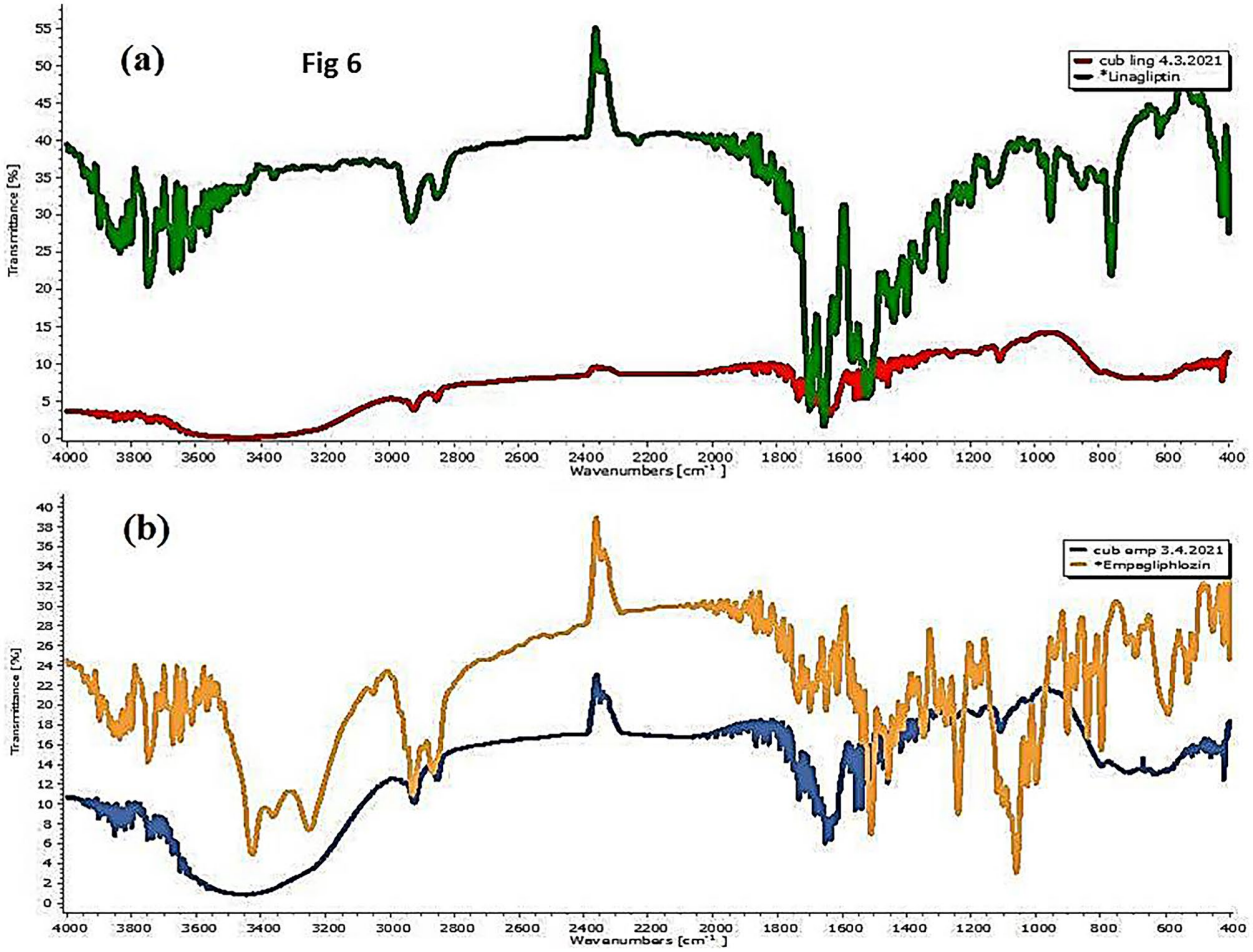
FTIR spectrum of **a** LCs and its components (including linagliptin, IPM, phosphatidylcholine, and Kolliphor RH40). **b** ECs and its components (including empagliflozin, IPM, phosphatidylcholine, and Kolliphore RH40)

FTIR spectrum (Fig. 6a, b) demonstrated that the characteristic absorption bands of Lin (1655 cm^−1^ (C = O stretching), 1517 cm^−1^ (C = N stretching), 1286 cm^−1^ (C–N stretching), and 763 cm^−1^ (C≡C bending)) and empagliflozin (3423 cm^−1^ (O–H stretching), 3250 cm^−1^ (aromatic C–H stretching), 797.57 cm^−1^ (C–CL stretching), and 999.68 cm^−1^ (C–O stretching)) disappeared, while the absorption bands completely matched with IPM, phosphatidylcholine, and Kolliphor RH40 absorption bands indicating the complete inclusion of linagliptin and empagliflozin inside the prepared cubosomes.

### Preparation of chitosan-PVP platform

To achieve effective buccal administration, it is important to design the buccal delivery system in a way to overcome buccal delivery challenges (e.g., salivation, buccal enzymatic degradation, and low contact period with mucosa) and improve the amount of drug permeated through buccal mucosa [37]. The chitosan-PVP platform of 4cm^2^ (containing 5 mg linagliptin and 10 mg empagliflozin) was efficiently prepared with the inclusion of optimized formulations LCs (F3) and ECs (F4).

The investigational values were suited to 2^2^ factorial designs; four formulae of the chitosan-PVP platform (LECF) were prepared and evaluated with respect to the desired criterion. The following polynomial equations were developed:

**Table:** 

Drug content of linagliptin	= + 69.24 + 3.33A − 1.05B − 9.02AB	(9)
Initial release of linagliptin	= + 1.94 + 0.30A + 0.43B − 0.092AB	(10)
Release (Q_24 h_) of linagliptin	= + 44.63 + 5.62A + 0.53B − 0.58AB	(11)
Drug content of empagliflozin	= + 86.56 + 3.71A + 0.57B − 9.57AB	(12)
Initial release of empagliflozin	= + 1.62 + 0.54A + 0.076B − 0.12AB	(13)
Release (Q_24 h_) of empagliflozin	= 51.00 + 5.84A + 0.45B + 2.14AB	(14)

### Characterization of chitosan-PVP platform (LECF)

All LECF formulations possessed a neutral pH (Table 6). Chitosan-PVP platform (LECF) indicated good mechanical strength and did not reveal any cracks after folding it for > 300 times for all platforms [38]. As seen in Table 6, the drug content of linagliptin and empagliflozin was performed for all chitosan-PVP platform formulations. The drug content was assessed using HPLC. The drug content values varied significantly between different LECFs (Table 6). The content of linagliptin and empagliflozin in chitosan-PVP platform formulations was demonstrated to be higher than that assayed in LC (F3) and EC (F4) systems (Table 5). This could be due to the fact that some of the drug amounts were adsorbed onto the surface of the cubosomes or entrapped within the aqueous channels of cubosomal nanoparticles. These situations might favor the rapid leakage of the drug from the aqueous channels to the surrounding aqueous phase during centrifugation [39]. The chitosan-PVP platform including powdered drugs (prepared by adding a definite amount of 5 mg linagliptin and 10 mg empagliflozin) has a drug content of 84.35% ± 1.22 and 78.47% ± 1.26 for linagliptin and empagliflozin, respectively. The results of the drug content of the chitosan-PVP platform including powdered drugs were used as control to reflect the effectiveness of using cubosomes in chitosan-PVP platform formulation. Using cubosomes resulted in increasing the empagliflozin drug content to 99.44% ± 0.35. Chitosan concentration could play an important role in drug content results. This was confirmed by the highest positive coefficient of chitosan concentration in Eqs. (9) and (12). The drug content percent of linagliptin and empagliflozin in a chitosan-PVP platform (LECF) containing 2% chitosan was higher than that containing 1% chitosan and similar to the results of a chitosan-PVP platform including powdered drugs. As the presence of chitosan caused a good solubilizing and/or wetting effect on linagliptin and empagliflozin respectively; increasing its concentration from 1 to 2% eventually showed higher drug content in the system [40].

**Table 6:** Characterization of chitosan-PVP platforms

**Formula**	**Thickness**	**Weight**	**Surface pH**	**Lin drug content (%)**	**Emp drug content (%)**	**Tensile strength** **(g/cm**^**2**^**)**	**Bioadhesive bond strength** **(N m**^**−2**^**)**
Plain	0.19 ± 0.01	225.43 ± 3.00	4.71 ± 0.30	–	–	20.75 ± 0.71	63.77 ± 3.5
Drug	0.52 ± 0.00	254.24 ± 8.35	6.03 ± 0.03	84.35 ± 1.22	78.47 ± 1.26	13.13 ± 0.88	38.01 ± 1.73
C_1_ (2% chitosan, 75 mg PVP-K30)	0.72 ± 0.01	435.94 ± 26.8	5.48 ± 0.04	81.88 ± 1.56	99.44 ± 0.35	21.50 ± 0.35	64.99 ± 5.2
C_2_ (2% chitosan, 150 mg PVP-K30)	0.87 ± 0.00	455.55 ± 3.67	5.39 ± 0.01	62.51 ± 0.06	81.00 0.58	21.00 ± 0.71	45.37 ± 5.2
C_3_ (1% chitosan, 75 mg PVP-K30)	0.66 ± 0.06	380.03 ± 10.9	5.71 ± 0.04	57.56 ± 0.71	72.22 ± 0.88	22.25 ± 0.35	47.82 ± 1.73
C_4_ (1% chitosan, 150 mg PVP-K30)	0.93 ± 0.00	426.79 ± 16.94	5.67 ± 0.06	73.82 ± 0.14	92.35 ± 1.28	21.5 ± 0.35	41.69 ± 3.5

#### Mucoadhesion strength

The higher concentration of chitosan showed higher mucoadhesion properties and higher bond strength with mucin due to the electrostatic bond [41]. But the mucoadhesive strength decreased with increasing PVP K-30 concentration due to its hydrophilic nature that loosens the mucoadhesion strength with the buccal mucosa [15].

#### Tensile strength, percent elongation, and swelling index (SI) measurement

**Fig. 7:**
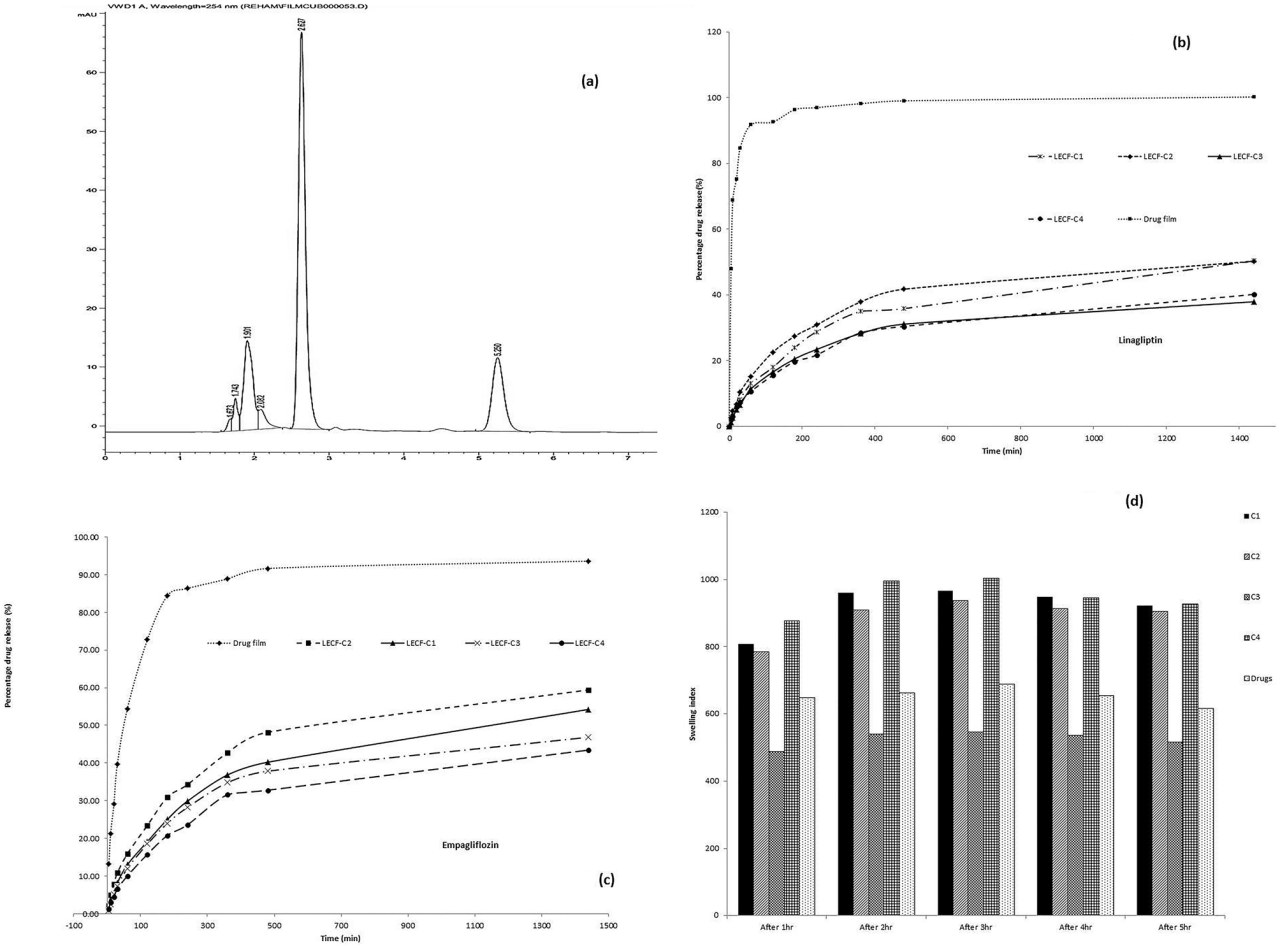
**a** Chromatographic assay of linagliptin and empagliflozin from film. **b** In-vitro release of linagliptin from LECF and the platform-containing powdered drugs. **c** In-vitro release of empagliflozin from LECF and the platform-containing powdered drugs. **d** Swelling index (SI) measurement of the LECF

SI of the LECFs (as shown in Fig. 7d) reached its maximum value within 3 h for all LECFs and then diminished due to platform erosion [42]. The platform-containing free powdered drugs (DF) showed a lower SI rather than that containing LCs and ECs at the same time point. The result of SI was related to the results of tensile strength measurement. This revealed that the addition of cubosomes (LCs and ECs) disturbed the intermolecular hydrogen bond formation between polymers, facilitating water diffusion*,* increasing the SI, and enhancing the motion and elasticity of LECF [42].

#### In-vitro release study

Results were shown in Fig. 7b, c. DF showed a higher release extent compared to LECFs. The LECF release mechanism involved, first, water diffusion, loosening of polymer chains, swelling, and erosion of the platform. Then, the drugs were released from the prepared cubosomes (LCs and ECs) [42]. Whereas DF release involved only water diffusion, loosening of polymer chains, swelling, and erosion of the platform and release of high drug concentration within 60 min (91.7% and 54.4% for Lin and Emp, respectively), the rapid release of drugs from DF did not fit the purpose of our study where the release of linagliptin is preferred to be in a continuous manner from different LECFs to resolve the non-linear pharmacokinetics resulting from its slow dissociation from the enzyme DPP-4 [9]. The drug release initially and after 24 h were significantly varied between different LECFs. Increased chitosan and PVP K-30 concentration led to a significant rise in linagliptin and empagliflozin release.

### Optimization of the prepared LECF

The optimized LECF (C1) system (prepared by 2% chitosan, 70 mg PVP K30) was selected with the aid of Design-Expert software in accordance to the desired criterion.

### Examination of LECF (C1) by scanning electron microscope

The morphology of the optimized LECF (C1) was investigated by SEM (Fig. 5c). SEM images indicated well-distributed LCs and ECs which were successfully enclosed within a partially smooth structure of the platforms.

### Ex vivo permeation study

The ex vivo permeation study was accomplished across sheep buccal mucosa for LECF (C1) and DF. Table 7 demonstrates the permeation data. The Lin and Emp permeation profiles from DF and from LECF displayed a lag time, as Lin and Emp belong to class III in the BCS classification characterized by low permeability [6]. However, the lag time from LECF is half that of DF. Moreover, the permeation extent of Lin and Emp from LECF was greater than the DF. The presence of LCs and ECs in LECF (C1) apparently increased their permeation through the paracellular and transcellular routes [43]. The presence of LCs and ECs in LECF (C1) could improve the paracellular transport through their nano-size and could improve the transcellular transport and drug solubility because phospholipids and non-ionic surfactants (Kolliphor RH40) act as permeation enhancers [43, 44]. Therefore, formulating a chitosan-PVP platform loaded with LCs and ECs greatly contributes to the permeation enhancement of the two drugs.

**Table 7:** Permeation data parameters

**Formulation/drug**	***R***^**2**^	**Flux (JSS)** **(µg/cm**^**2**^**/min)**	***P***_**app**_ **(cm**^**2**^**/min)**	**D** **(cm**^**2**^**/min)**	**Lag time** **(TL)** **(min)**	**Permeated at the end of 8 h (%)**
LECF (C1) (linagliptin)	0.9815	0.4938	9.9 × 10^−5^	1.5 × 10^−3^	13	33.85%
Drug film (Lin)	0.9951	0.0469	9.4 × 10^−6^	7.7 × 10^−4^	25	2.85%
LECF (C1) (empagliflozin)	0.9677	0.8002	8 × 10^−5^	4.8 × 10^−3^	4	28.7%
Drug film (Emp)	0.8876	0.1789	1.8 × 10^−5^	2.4 × 10^−3^	8	5%

### Pharmacokinetic study

**Fig. 8:**
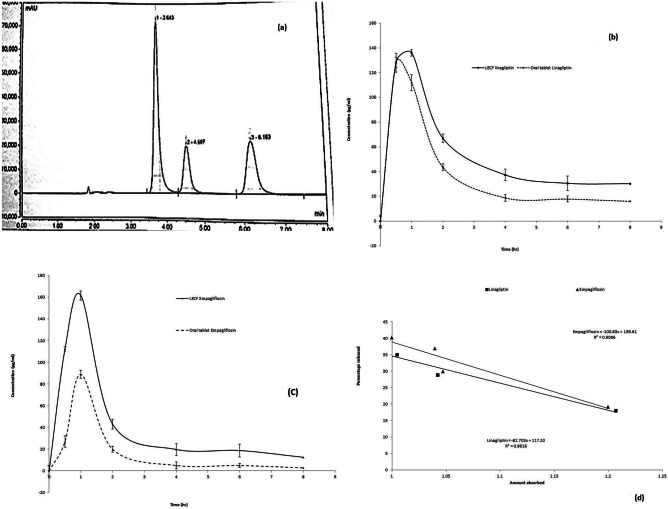
**a** Chromatographic separation of linagliptin, IS, and empagliflozin, respectively, in the in-vivo study. **b** Comparative pharmacokinetics studies of linagliptin of optimized buccal LECF (C1) and oral tablet market ± SD. **c** Comparative pharmacokinetics studies of empagliflozin of optimized buccal LECF (C1) and oral tablet market ± SD. **d** In-vitro/in-vivo correlation of linagliptin and empagliflozin from the optimized LECF (C1)

The analytical assay of drugs within rabbit plasma was validated with respect to USP general chapter validation requirements (1225). The effective assay of Emp and Lin in rabbit plasma was done according to Hammad et al.’s method [16] (Fig. 8a). The method showed good linearity, accuracy, acceptable precision, and inter- and intra-day reproducibility. The LOD (lowest detection limit) in rabbit plasma was 44 ng/mL for drugs. The mean plasma concentration–time curves after administration of buccal LECF (C1) and oral tablets are illustrated in Fig. 8b, c. The pharmacokinetic study results regarding *C*_max_, *T*_max_, *K*_el_, *T*_1/2_, AUC_(0–24)_, and AUC_(0–∞)_ are illustrated in Table 8. The values of *C*_max_, AUC_(0–24)_, and AUC_(0–∞)_ after buccal administration of LECF (C1) were significantly greater than the oral tablet (*p* < 0.05) when analyzed by SPSS software which indicates a higher absorption rate and bioavailability. The investigated *T*_max_ value for Lin was increased to reach 1 h, while it was 0.5 h for the oral tablet, which is identical to that previously published in its European Public Assessment Report [5]. The rise in *T*_max_ of Lin to reach 1 h from LECF (C1) confirmed the continuous efflux of Lin from the LECF which could resolve its non-linear pharmacokinetics and maximize its dose effectiveness. The investigated *T*_max_ value of Emp was not changed. The value of AUC_(0–∞)_ for both drugs was higher than the value of their respective oral tablets (Table 8). This result confirmed the improved permeability and bioavailability of both drugs when they were formulated as dual cubosomes and enclosed within the chitosan-PVP platform for buccal administration. These strategies could greatly contribute to the enhancement of their bioavailability and clinical effectiveness.

**Table 8:** *C*_max_, *T*_max_, *K*_el_, *T*_1/2_, AUC_(0–24)_, and AUC_(0–∞)_ data from buccal LECF (C1) and oral marketed tablet

**Pharmacokinetic parameter**	**Lin**		**Emp**	
	**LECF (C1)**	**Oral tablet**	**LECF (C1)**	**Oral tablet**
***C***_max_ (μg/ml)	136.81 ± 2.71	127.94 ± 3.19	161.54 ± 4.78	88.85 ± 4.01
***T***_max_ (h)	1 h	0.5 h	1 h	1 h
AUC_(0–24)_ (μg.h/ml)	674.78 ± 37.27	429.61 ± 13.47	430.87 ± 29.07	154.54 ± 7.73
AUC_(0–∞)_ (μg.h/ml)	814.86 ± 37.3	485.01 ± 14.76	469.37 ± 29.1	161.5 ± 8.47
***K***_el_ (h^−1^)	0.2 ± 0.009	0.3 ± 0.01	0.3 ± 0.022	0.4 ± 0.026
***T***_1/2_ (h)	3.23 ± 0.15	2.41 ± 0.08	2.14 ± 0.15	1.75 ± 0.11

### Development of the in-vitro/in-vivo correlation model

Correlation among in-vitro released data and in-vivo data for LECF (C1) ensured the simulation of the in-vitro released data and in-vivo results (Fig. 8d). The correlation coefficients (*R*^2^) obtained by mean of linear regression were 0.9616 and 0.9086 (for linagliptin and empagliflozin, respectively) indicating an excellent linear relationship when correlating them at the same time points [17].

#### Buccal histopathology

**Fig. 9:**
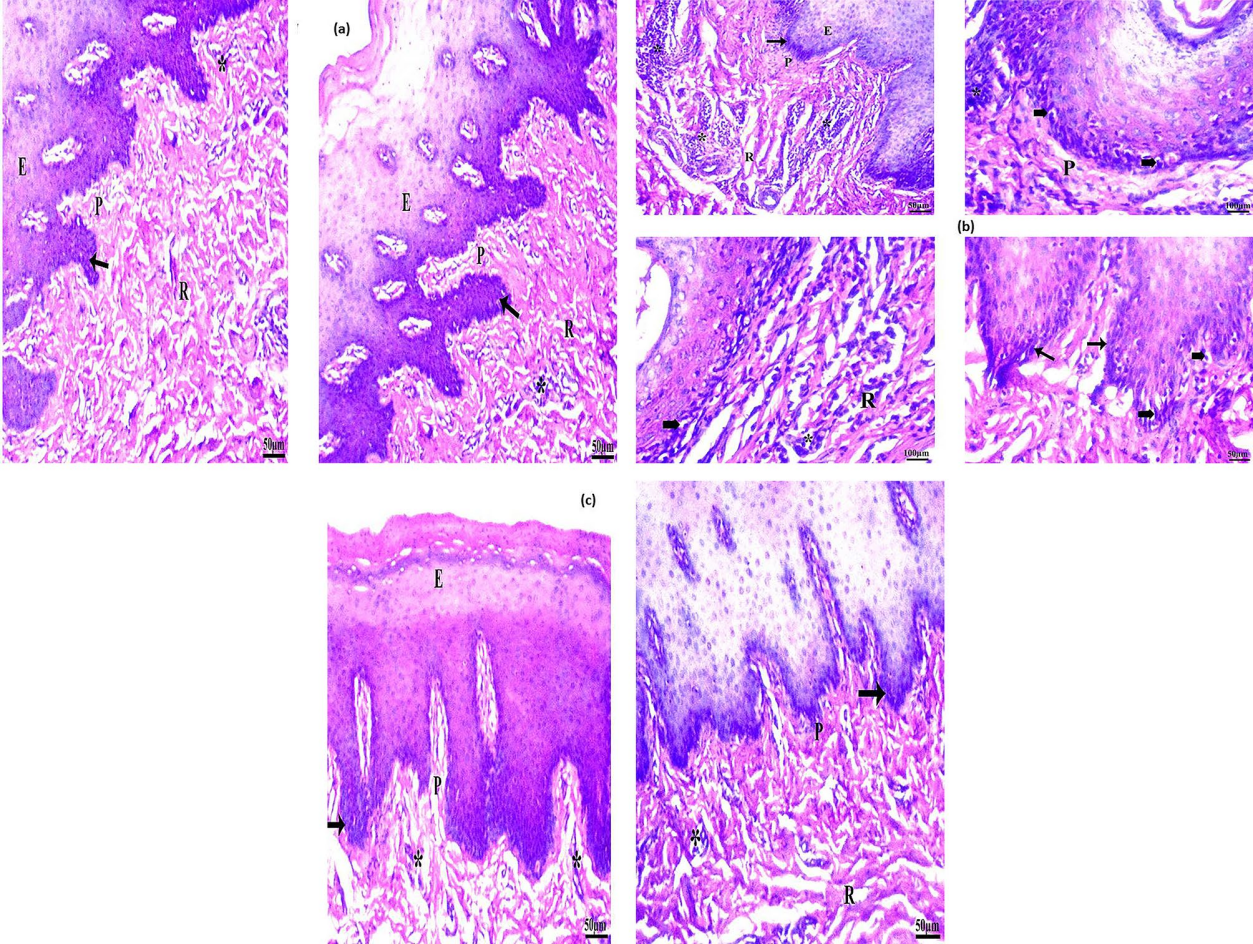
**a** Photomicrograph of sheep buccal mucosa of negative control treated with PBS pH 6.8 showed keratinized stratified squamous epithelium (E) with usual, broad, and short rete pegs (arrow). The lamina propria indicated the papillary region (p) and reticular (R), well-coordinated collagen fibers. Normal appearance of little inflammatory cells is indicated as “*”. **b** Photomicrograph of sheep buccal mucosa of positive control treated with isopropyl alcohol demonstrated distinct damaged cells with pyknotic nuclei (bold arrow), flattening, or shortening of rete ridge (arrow), extensive and severe inflammatory cells indicated as “*” infiltration in the lamina propria and disturbance of collagen fibers arrangement (R) with wide gaps indicated as “**”. **c** Photomicrograph of sheep buccal mucosa of the optimized LECF showed well-developed rete pegs (arrow)

Buccal histopathology is advantageous to examine the safe effects of LECF on the sheep buccal mucosa integrity [16]. Three pieces of sheep buccal mucosa were used in this study. One was immersed in phosphate-buffered saline pH 6.8 (negative control), the second was treated with LECF, and the third was treated with isopropyl alcohol (positive control) (as shown in Fig. 9). Positive control demonstrated distinct damage with extensive and severe inflammatory cell infiltration and disturbance of collagen fiber arrangement. The piece treated with the optimized LECF revealed that there were no significant changes observed in the histological pattern of the mucosa. This indicated the safety of LECF for buccal administration.

## Conclusion

The goal of this study was to formulate a novel chitosan-PVP platform embedded cubosomes including a synergistic combination of Lin and Emp to augment their bioavailability, increase patient satisfaction, and maximize their clinical effectiveness in the management of unresponsive hyperglycemia in type II diabetes mellitus with associated cardiovascular disease side effect. Dual cubosomes loaded with Lin and Emp (LCs and ECs) were successfully prepared and optimized. The optimized LCs and ECs were enclosed within the chitosan-PVP platform. The optimized LECF (C1) showed continuous efflux of Lin which resolved its non-linear pharmacokinetic profile and an increase in Emp permeation. The overall formulation could successfully contribute to a better performance in the treatment of persistent diabetes through a dual enhancement of Lin and Emp bioavailability.

## Data Availability

Additional data and materials will be available upon request.
